# Supplementary material for: Holistic analysis of a gliding arc discharge using 3D tomography and single-shot fluorescence lifetime imaging
Source: Commun Eng. 2024 Jul 24;3:103. doi: 10.1038/s44172-024-00250-z (PMC11269738; doi:10.1038/s44172-024-00250-z)
Supplement: Supplementary file 2 — Description of Additional Supplementary Files [file 44172_2024_250_MOESM2_ESM.pdf]

Description of Additional Supplemntary Files

File name: Supplemnatary Material 1

File description: 3D Renditions of Tomography and Fluorescence Imaging
